# Supplementary material for: A Critical View of the Application of the APEX Software (Aqueous Photochemistry of Environmentally-Occurring Xenobiotics) to Predict Photoreaction Kinetics in Surface Freshwaters
Source: Molecules. 2019 Dec 18;25(1):9. doi: 10.3390/molecules25010009 (PMC7017383; doi:10.3390/molecules25010009)
Supplement: Supplementary file 1 [file molecules-25-00009-s001.zip › 3.2.4_gcc-4.4.0.zip!/3.2.4_gcc-4.4.0/3.2.4_gcc-4.4.0/doc/octave/html/interpreter/Creating-Diagonal-Matrices.html]

Creating Diagonal Matrices - Untitled


Next: Creating Permutation Matrices,
Up: Basic Usage


---

#### 20.1.1 Creating Diagonal Matrices

The most common and easiest way to create a diagonal matrix is using the built-in
function diag. The expression `diag (v)`, with v a vector,
will create a square diagonal matrix with elements on the main diagonal given
by the elements of v, and size equal to the length of v.
`diag (v, m, n)` can be used to construct a rectangular diagonal matrix.
The result of these expressions will be a special diagonal matrix object, rather
than a general matrix object.

Diagonal matrix with unit elements can be created using eye.
Some other built-in functions can also return diagonal matrices. Examples include
balance or inv.

Example:

```
       diag (1:4)
     =>
     Diagonal Matrix
     
        1   0   0   0
        0   2   0   0
        0   0   3   0
        0   0   0   4
     
       diag(1:3,5,3)
     
     =>
     Diagonal Matrix
     
        1   0   0
        0   2   0
        0   0   3
        0   0   0
        0   0   0
```
